# Targeting Cathepsin C in Cancer Metastasis: Protease Network Activation, Inflammatory Crosstalk, and Therapeutic Opportunities

**DOI:** 10.3390/ijms27125369

**Published:** 2026-06-14

**Authors:** Yahui Liu, Liangyu Hao, Lixiang Zheng

**Affiliations:** College of Life Sciences, Jiangxi University of Traditional Chinese Medicine, Nanchang 330004, China; liuyahui@jxutcm.edu.cn (Y.L.); haoliangyu@jxutcm.edu.cn (L.H.)

**Keywords:** cathepsin C, metastasis, therapeutic target, protease network, inflammatory crosstalk

## Abstract

Cathepsin C (CTSC), also known as dipeptidyl peptidase I, is an upstream activator of serine protease networks that may promote metastatic progression through inflammatory amplification and microenvironmental remodeling. Increasing evidence suggests that CTSC contributes to cancer progression not simply as an overexpressed lysosomal protease, but as a context-dependent regulator of metastatic traits. This review summarizes the structure, maturation, and biological functions of CTSC, with emphasis on its protease-activating capacity and its links to tumor-associated inflammation. Current evidence connecting CTSC to epithelial–mesenchymal transition, extracellular matrix remodeling, neutrophil extracellular trap formation, and immune microenvironment reprogramming is then synthesized across hepatocellular carcinoma, renal cell carcinoma, breast cancer, colorectal cancer, non-small-cell lung cancer, and glioma. Available data most strongly support a pro-metastatic role for CTSC in breast cancer and colorectal cancer, whereas evidence in several other malignancies remains predominantly preclinical and mechanistically incomplete. Importantly, CTSC is better viewed as a targetable protease network hub than as a universal pan-cancer metastatic driver. The biomarker potential and therapeutic relevance of CTSC are also evaluated, with particular attention to the opportunities and limitations of current DPP-1/CTSC inhibitors and the need for tumor-specific translational strategies. Overall, CTSC represents a promising but still incompletely validated target in oncology, and future work should prioritize tissue-specific dependency, biomarker qualification, and rational combination approaches.

## 1. Introduction

Cathepsin C (CTSC), also known as dipeptidyl peptidase I, is a lysosomal cysteine protease best known for activating multiple serine proteases involved in inflammation and immune defense [1]. Unlike many other cysteine cathepsins that primarily function as degradative proteases, CTSC occupies a unique upstream position within protease activation cascades because it converts numerous serine protease zymogens into their active forms [2]. Through this role, CTSC can regulate inflammatory amplification, extracellular matrix remodeling, and immune cell effector functions.

Within the tumor microenvironment, CTSC is highly enriched in inflammatory and immune-associated cell populations, including neutrophils, macrophages, mast cells, cytotoxic T lymphocytes, and natural killer cells [3]. This cellular distribution is biologically important because these immune populations actively participate in inflammatory signaling, matrix remodeling, immune suppression, and metastatic niche conditioning [4]. As a result, CTSC is increasingly recognized not simply as a tumor-associated protease, but as a mediator linking inflammatory crosstalk to metastatic progression.

Emerging evidence suggests that CTSC contributes to cancer metastasis through several interconnected mechanisms, including neutrophil serine protease activation, epithelial–mesenchymal transition (EMT), extracellular matrix remodeling, neutrophil extracellular trap (NET) formation, and immune microenvironment reprogramming [5]. In particular, CTSC-dependent activation of neutrophil elastase, cathepsin G, and proteinase 3 may amplify inflammatory feed-forward signaling and facilitate pre-metastatic niche formation in distant organs such as the lung [6]. Recent studies have further demonstrated that CTSC promotes breast cancer lung metastasis by enhancing neutrophil recruitment and NET formation, thereby establishing a metastasis-permissive inflammatory microenvironment [7].

Importantly, CTSC should not be interpreted in isolation from broader protease systems. Increasing evidence suggests that metastatic progression is orchestrated by interconnected protease networks involving multiple cathepsin family members and inflammation-associated proteases [8]. Among these, cathepsin B (CTSB) has long been implicated in extracellular matrix degradation, invasion, angiogenesis, and metastatic dissemination [9]. While CTSB primarily functions as a direct matrix-remodeling protease, CTSC appears to act more prominently as an upstream regulator of protease network activation and inflammatory amplification [10]. These proteases may therefore cooperate within layered proteolytic circuits that shape tumor–stromal communication, immune cell behavior, and metastatic organotropism.

Nevertheless, the literature surrounding CTSC remains fragmented across tumor types, and CTSC is often discussed either as a generic cathepsin family member or as an isolated observation in single cancers. Moreover, the biological relevance of CTSC appears highly context dependent, varying according to tumor type, immune composition, and metastatic organ microenvironment [5]. This review therefore focuses on CTSC as a context-dependent and targetable protease network hub and critically evaluates the current evidence linking CTSC to tumor-associated inflammation, metastatic niche remodeling, and cancer progression. Particular attention is given to the translational opportunities and limitations of CTSC-targeted therapeutic strategies across different tumor settings.

### Literature Search Strategy

The literature for this narrative review was identified through PubMed, Web of Science, and Scopus up to April 2026 using combinations of the terms “cathepsin C”, “dipeptidyl peptidase I”, “DPP-1”, “cancer”, “metastasis”, “tumor microenvironment”, “neutrophils”, “NETs”, “EMT”, “biomarker”, and “inhibitor”. Searches were supplemented by manual screening of references from relevant mechanistic studies and high-quality reviews on CTSC biology, serine-protease activation, neutrophil extracellular traps, and protease-targeted therapy.

Articles were considered eligible when they met at least one of the following criteria: (i) direct investigation of CTSC/DPP-1 expression, function, or inhibition in cancer; (ii) mechanistic analysis of CTSC-linked downstream processes, including neutrophil serine protease activation, NET formation, EMT, myeloid cell remodeling, or metastatic niche formation; or (iii) translational or clinical relevance to CTSC inhibitors, protease network biology, or metastasis-associated inflammatory microenvironments. Peer-reviewed original studies, translational reports, clinical investigations, and mechanistically relevant reviews were prioritized. Studies were excluded if they only discussed cathepsins in general without distinguishable relevance to CTSC, lacked a clear relationship to cancer progression or metastasis, were duplicate records, or provided only conference-level or non-peer-reviewed information.

Evidence was interpreted according to biological relevance and strength of inference. Highest weight was given to studies combining CTSC perturbation with functional metastatic or invasive phenotypes, especially when supported by in vivo or clinically annotated data. Cell-based studies, bioinformatic associations, and single-cohort prognostic analyses were treated as hypothesis-generating unless supported by mechanistic validation. Throughout the review, direct CTSC-specific evidence was distinguished from broader NET-, neutrophil-, or tumor microenvironment-related evidence, and correlative findings were not interpreted as proof of causality.

## 2. Structural and Functional Features of CTSC

### 2.1. CTSC Within the Cysteine Cathepsin Family

Cathepsin C (CTSC), also known as dipeptidyl peptidase I (DPPI), belongs to the cysteine cathepsin family, a group of lysosomal proteases primarily classified within the papain-like C1 protease superfamily [1,9]. Cysteine cathepsins constitute one of the major proteolytic systems involved in intracellular protein turnover, lysosomal degradation, immune regulation, extracellular matrix remodeling, and inflammatory signaling [1]. Among this family, cathepsins B (CTSB), L (CTSL), K (CTSK), S (CTSS), and C (CTSC) are among the most extensively studied members in cancer and inflammatory diseases [1,9].

Although many cysteine cathepsins share conserved catalytic mechanisms and lysosomal localization, CTSC possesses several unique structural and functional characteristics that distinguish it from other family members [10,11]. CTSB and CTSL primarily function as endopeptidases that directly degrade intracellular and extracellular substrates, whereas CTSK is specialized for collagen degradation during bone remodeling [1]. In contrast, CTSC functions predominantly as an upstream protease activator rather than a bulk degradative protease [2,3]. This “protease-activating function” represents the defining biological feature of CTSC [10].

Unlike most cathepsins, CTSC preferentially removes N-terminal dipeptides from substrate proteins and plays an essential role in converting multiple serine protease zymogens into their mature active forms [3,12]. Through this mechanism, CTSC regulates activation of neutrophil elastase (NE), cathepsin G (CatG), proteinase 3 (PR3), neutrophil serine protease 4 (NSP4), mast cell chymase and tryptase, and cytotoxic lymphocyte granzymes [3,12,13]. Consequently, CTSC acts as a central upstream regulator of inflammatory protease networks rather than merely a terminal degradative enzyme [2,10].

This upstream positioning gives CTSC disproportionate biological influence within inflammatory and tumor-associated protease cascades [5,10]. Even modest alterations in CTSC activity may substantially amplify downstream proteolytic signaling, extracellular matrix remodeling, cytokine activation, and immune-cell crosstalk [5]. This network-amplifying property may explain why CTSC contributes to cancer progression despite not always being among the most highly expressed cathepsins within tumor tissues [10].

### 2.2. Structural Organization and Catalytic Features of CTSC

Human CTSC is synthesized as an inactive preproenzyme composed of several distinct structural regions, including an N-terminal signal peptide, a propeptide region, an exclusion domain, and the catalytic domain that is subsequently processed into heavy and light chains [14,15,16].

The signal peptide directs newly synthesized CTSC into the endoplasmic reticulum (ER), where early folding and glycosylation events occur [15,16]. Following signal peptide removal, the propeptide functions as an intramolecular chaperone that stabilizes the immature enzyme and prevents premature catalytic activation during intracellular trafficking [15].

One of the most distinctive structural features of CTSC is the exclusion domain, which is absent in many other cysteine cathepsins [10,14]. The exclusion domain partially occludes the active-site cleft and sterically restricts substrate access beyond the S2 binding pocket [14]. This unique structural arrangement determines the dipeptidyl peptidase activity of CTSC by allowing cleavage of only N-terminal dipeptides rather than longer peptide fragments [10,14]. Importantly, the exclusion domain is also critical for selective activation of granule-associated serine protease zymogens [2,3].

The catalytic region of CTSC adopts the classical papain-like fold characteristic of C1 cysteine proteases and contains the conserved catalytic triad composed of cysteine, histidine, and asparagine residues [1,14]. These residues cooperate to mediate nucleophilic peptide bond hydrolysis under acidic lysosomal conditions [1].

Following proteolytic maturation, the catalytic domain is separated into heavy and light chains that remain connected through disulfide interactions and noncovalent associations [15,16]. Mature CTSC ultimately assembles into a homotetrameric complex [14,15,16], a structural feature required for optimal enzymatic activity and substrate processing [11,14].

Importantly, the structural organization of CTSC directly underlies its biological specialization. While other cathepsins primarily function as degradative proteases, the exclusion-domain-dependent substrate restriction enables CTSC to function as a highly selective protease activator, thereby linking lysosomal proteolysis to inflammatory signaling and immune-cell effector activation [3,10]. From a cancer biology perspective, this structural specialization helps explain why CTSC may influence metastatic progression indirectly through downstream protease network activation rather than through direct matrix degradation alone [5,10]. Therefore, the structural features of CTSC provide a mechanistic basis for interpreting it as an upstream regulatory node within tumor-associated inflammatory protease circuits, rather than as a conventional degradative cathepsin [8,10].

### 2.3. Biosynthesis, Maturation, and Lysosomal Activation of CTSC

CTSC undergoes a complex multistep maturation process involving ER entry, N-linked glycosylation, mannose-6-phosphate-dependent lysosomal targeting, proteolytic processing, tetramer assembly, and lysosomal activation [15,16]. This maturation sequence is substantially more elaborate than that of many other cysteine cathepsins and is essential for full enzymatic competence [10,16].

Newly synthesized CTSC enters the endoplasmic reticulum through its signal peptide, where initial N-linked glycosylation and protein folding occur [15,16]. Glycosylation contributes not only to structural stability but also to intracellular trafficking and lysosomal targeting [15]. Properly folded CTSC is subsequently transported through the Golgi apparatus and directed toward lysosomes via mannose-6-phosphate-dependent trafficking pathways [17].

Immature CTSC is synthesized as an inactive zymogen and requires sequential proteolytic processing for activation [15,16]. Several upstream lysosomal endopeptidases, including cathepsins L and S, have been implicated in cleavage of the CTSC proregion [15,16]. This processing removes inhibitory segments and generates the mature heavy- and light-chain architecture characteristic of active CTSC [16].

After proteolytic maturation, CTSC monomers assemble into homotetrameric complexes within the acidic lysosomal environment [14,15,16]. Tetramer formation is critical for enzymatic stability and catalytic activity, because isolated catalytic domains exhibit altered substrate accessibility and reduced physiological functionality [14]. Lysosomal acidification further optimizes catalytic efficiency and maintains conformational stability of the mature enzyme [1].

Importantly, CTSC maturation is tightly linked to immune cell differentiation and granule biogenesis [2,3]. In neutrophils, mast cells, and cytotoxic lymphocytes, mature CTSC colocalizes with granule-associated serine protease precursors and mediates their activation during granule maturation [3,12,13]. Therefore, defects in CTSC processing or trafficking can broadly impair inflammatory protease networks [2].

The biological importance of proper CTSC maturation is further illustrated by Papillon-Lefèvre syndrome, a hereditary disorder caused by loss-of-function CTSC mutations [2,10]. Patients exhibit severe periodontitis, recurrent infections, and impaired neutrophil serine protease activation, highlighting the central role of CTSC in immune protease homeostasis [2]. Among currently characterized post-translational events, N-linked glycosylation and mannose-6-phosphate-dependent lysosomal targeting appear to be the best-established modifications relevant to CTSC maturation and intracellular trafficking. In contrast, CTSC-specific functional evidence for other PTMs, such as phosphorylation, acetylation, or ubiquitination, remains limited. Therefore, in the context of CTSC biology, post-translational regulation is best discussed through glycosylation-dependent folding, lysosomal sorting, and proteolytic maturation rather than through broadly generalized PTM categories.

**Figure 1:**
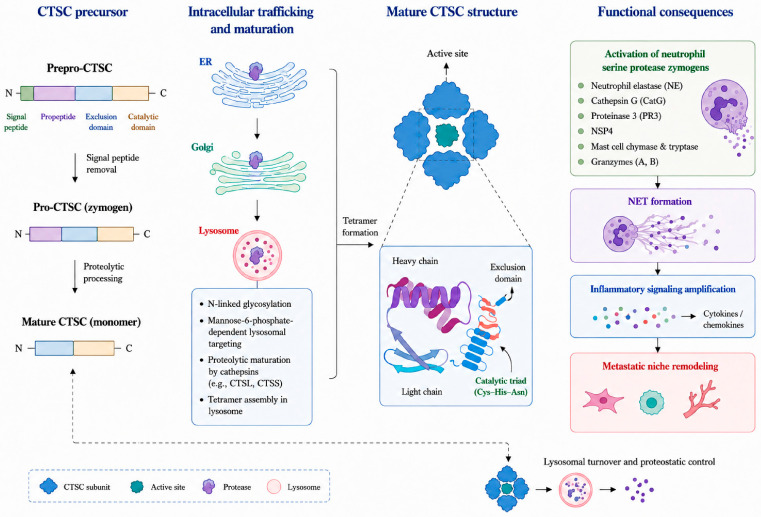
Structure, maturation, and functional activation of cathepsin C (CTSC).

Taken together, these processes indicate that CTSC maturation depends on coordinated intracellular trafficking, glycosylation-dependent lysosomal targeting, proteolytic processing, lysosomal activation, and tetramer assembly, ultimately enabling downstream protease network activation and inflammatory signaling (Figure 1). In tumor-associated inflammatory microenvironments, such maturation control may be particularly relevant because neutrophils, macrophages, mast cells, and cytotoxic lymphocytes can provide cellular reservoirs in which mature CTSC supports downstream effector protease activation [3,4,5]. Thus, altered CTSC maturation or lysosomal routing may not simply affect enzyme abundance, but may reshape the intensity and location of protease-driven immune crosstalk during metastatic niche formation [5,8,10,18].

CTSC is synthesized as an inactive preproenzyme containing a signal peptide, propeptide, exclusion domain, and catalytic domain. Following signal peptide removal, pro-CTSC undergoes intracellular trafficking through the endoplasmic reticulum (ER), Golgi apparatus, and lysosomal compartments, where N-linked glycosylation, mannose-6-phosphate-dependent lysosomal targeting, and proteolytic processing contribute to maturation. Mature CTSC assembles into a homotetrameric complex composed of heavy and light chains and containing the characteristic exclusion domain and catalytic triad (Cys-His-Asn). Active CTSC subsequently mediates activation of multiple neutrophil and immune-cell serine proteases, including neutrophil elastase (NE), cathepsin G (CatG), proteinase 3 (PR3), NSP4, mast cell proteases, and granzymes. Downstream biological consequences include NET formation, inflammatory signaling amplification, and metastatic niche remodeling. CTSC activity is further regulated through lysosomal turnover and proteostatic control.

### 2.4. Protease-Activating Function and Biological Significance

The defining physiological function of CTSC is activation of granule-associated serine proteases involved in innate and adaptive immunity [3,12,13]. CTSC removes N-terminal dipeptides from inactive zymogens, thereby generating catalytically competent serine proteases [3].

Activated downstream proteases include neutrophil elastase, cathepsin G, proteinase 3, NSP4, mast cell chymase, tryptase, and lymphocyte granzymes [3,12,13]. These enzymes collectively regulate extracellular matrix degradation, cytokine maturation, chemokine processing, antimicrobial defense, and inflammatory amplification [2,3].

Because CTSC occupies an upstream regulatory position within this protease hierarchy, it functions as a molecular amplifier of inflammatory signaling [5,10]. CTSC-dependent activation of neutrophil serine proteases can promote extracellular matrix remodeling, endothelial injury, cytokine release, and neutrophil extracellular trap (NET) formation [2,5]. These downstream effects are increasingly recognized as important contributors to tumor progression and metastatic niche conditioning [5].

Importantly, CTSC should not be interpreted simply as an isolated lysosomal protease. Rather, it acts as a nodal regulator connecting lysosomal proteolysis, inflammatory signaling, immune-cell activation, and extracellular protease cascades [10]. This systems-level role distinguishes CTSC from many other cathepsins whose biological effects arise primarily through direct substrate degradation [1,10]. In metastatic cancer, this distinction is especially important because inhibition of CTSC could theoretically attenuate multiple downstream neutrophil- and myeloid-associated proteases at once, rather than blocking only a single terminal effector [5,8]. However, the biological consequence of such upstream inhibition is likely to vary across tumor types, depending on immune-cell composition, neutrophil dependence, and the dominant protease networks within each metastatic niche [5,8,19].

### 2.5. Lysosomal Turnover, Proteostatic Control, and Pathological Dysregulation of CTSC

CTSC proteostasis is controlled not by a single degradation pathway, but by a coordinated lysosomal life cycle that includes glycosylation-dependent trafficking, lysosomal delivery, proteolytic maturation, tetramer stabilization, substrate engagement, and lysosomal turnover [15,16,17,18]. After synthesis and mannose-6-phosphate-dependent sorting, pro-CTSC reaches lysosomal compartments where cathepsins L and S contribute to proregion processing and generation of mature heavy- and light-chain forms [15,16,17]. Mature CTSC is then maintained within acidic lysosomal or granule-associated compartments, where its stability and activity depend on proper lysosomal pH, balanced protease activity, and tetrameric assembly [14,15,16].

Under physiological conditions, this compartmentalized maturation-and-turnover system helps restrict CTSC activity to appropriate intracellular or granule-associated environments. Lysosomal turnover prevents excessive persistence of mature CTSC, whereas trafficking quality control and proteolytic processing ensure that incompletely matured or mislocalized enzyme does not inappropriately activate downstream protease cascades [1,10,18]. This regulation is particularly important in neutrophils, mast cells, and cytotoxic lymphocytes, where CTSC-dependent activation of granule-associated serine proteases directly shapes inflammatory and immune effector functions [2,3,12,13].

Dysregulation may occur at several points along this axis. Defective lysosomal trafficking or altered lysosomal acidification may impair normal maturation and compartmentalization of CTSC, whereas increased lysosomal biogenesis, inflammatory cell expansion, or granule remodeling may increase the pool of mature enzyme available for downstream serine protease activation [10,18]. In neutrophil-rich or myeloid-rich tumor microenvironments, this may amplify activation of neutrophil elastase, cathepsin G, and proteinase 3, thereby promoting extracellular matrix remodeling, cytokine processing, NET formation, and inflammatory feed-forward signaling [2,3,4,5].

In cancer, CTSC dysregulation should therefore be understood as part of a broader lysosomal and inflammatory protease-network imbalance rather than as an isolated increase in a single enzyme. Aberrant CTSC activity may exert biological effects disproportionate to its expression level because CTSC acts upstream of multiple serine proteases. Even modest changes in CTSC maturation, localization, or activity could therefore intensify downstream proteolytic signaling, immune-cell crosstalk, stromal remodeling, and metastatic niche conditioning [5,10]. This network-amplifying property provides a mechanistic basis for linking CTSC proteostasis to tumor-associated inflammation and metastatic progression.

However, direct evidence linking CTSC degradation kinetics to cancer metastasis remains limited. Most current support is indirect and derives from the established requirement of CTSC for granule-associated serine protease activation, the immune phenotype of CTSC loss-of-function disorders, and tumor studies showing that CTSC-dependent inflammatory protease networks can promote metastatic phenotypes [2,5,7,10]. Therefore, CTSC proteostatic dysregulation should presently be interpreted as a plausible mechanism connecting lysosomal remodeling to metastatic inflammation, rather than as a fully validated cancer-specific degradation pathway.

Overall, CTSC degradation and proteostatic regulation are best framed as part of a lysosome-centered maturation, activation, and turnover process. This framing avoids overstating poorly characterized degradation mechanisms while emphasizing the biologically important point that altered CTSC trafficking, maturation, or stability may reshape downstream inflammatory protease networks in pathological and tumor-associated microenvironments. From a translational standpoint, this also suggests that CTSC-targeted strategies should consider not only enzyme expression, but also maturation state, cellular source, lysosomal localization, and downstream protease activity when evaluating CTSC as a biomarker or therapeutic target [5,8,10].

## 3. CTSC as a Context-Dependent Driver of Metastatic Protease Networks

The pro-metastatic role of CTSC appears to arise from the convergence of several molecular and cellular programs rather than from a single linear pathway. As an upstream activator of neutrophil and immune-cell serine proteases, CTSC can amplify proteolytic and inflammatory cascades that favor extracellular matrix degradation and metastatic niche remodeling [5,8,19]. CTSC-dependent neutrophil activation may also facilitate NET formation and pro-metastatic inflammatory amplification [20]. Tumor-specific studies further link CTSC to cell-intrinsic invasive signaling in RCC, HCC, NSCLC, and glioma, as well as to CSF1-dependent MDSC/TAM accumulation in colorectal cancer [21,22,23,24,25].

### 3.1. Protease-Network Crosstalk and Amplification

Beyond its individual enzymatic activity, CTSC is better understood as an upstream amplifier within a broader protease network. The biological significance of CTSC does not primarily arise from direct bulk degradation of extracellular matrix components, but from its ability to convert multiple serine protease zymogens into active effectors, thereby initiating secondary proteolytic cascades and inflammatory feed-forward loops [10]. In this framework, CTSC links lysosomal protease biology to neutrophil serine protease activation, tissue injury, inflammatory signaling, and microenvironmental remodeling.

This network-level perspective is particularly relevant in cancer metastasis. Activated downstream proteases such as neutrophil elastase, cathepsin G, and proteinase 3 can modulate extracellular matrix integrity, cytokine maturation, chemokine activity, and tumor–stromal communication [3]. As a result, CTSC-dependent protease activation may not simply increase proteolysis in a quantitative sense, but may qualitatively reshape the metastatic niche by altering inflammatory tone, matrix composition, and immune-cell behavior.

Importantly, CTSC should be distinguished from other metastasis-associated proteases in terms of functional hierarchy. Cathepsin B (CTSB) is more commonly associated with direct extracellular matrix degradation, basement membrane remodeling, invasion, and angiogenesis [9,19]. Matrix metalloproteinases (MMPs), particularly MMP2 and MMP9, act mainly as extracellular matrix-remodeling enzymes that facilitate invasion, tissue barrier disruption, and metastatic dissemination [8,19]. Neutrophil elastase (NE), cathepsin G, and proteinase 3 function as downstream inflammatory serine protease effectors that can promote tissue remodeling, cytokine processing, endothelial injury, NET formation, and pro-metastatic inflammatory signaling [2,3,4,5]. By contrast, CTSC occupies a more upstream position because it regulates the activation of several of these immune cell serine proteases rather than acting only as a terminal matrix-degrading enzyme [3,10].

This distinction is biologically important because an upstream hub can influence multiple downstream proteolytic and inflammatory processes simultaneously. In CTSC-rich inflammatory microenvironments, enhanced CTSC activity may amplify neutrophil elastase-, cathepsin G-, and proteinase 3-dependent effects, which may then cooperate with CTSB- and MMP-mediated matrix remodeling to generate a permissive metastatic niche [5,8,19]. Therefore, CTSC should not be interpreted as functionally equivalent to CTSB or MMPs; rather, it may act as a regulatory connector linking lysosomal maturation, immune cell protease activation, extracellular proteolysis, and inflammatory crosstalk.

From a conceptual standpoint, this protease network model helps explain why CTSC may exert substantial biological effects even when its direct expression level is only moderately altered: upstream nodes can disproportionately influence downstream effector intensity. It also provides a rationale for selective therapeutic targeting, because inhibition of an upstream activator such as CTSC may attenuate several downstream metastasis-promoting pathways simultaneously [5]. Nevertheless, this hypothesis remains context-dependent and requires validation in tumor-specific systems.

An important unresolved issue is whether CTSC-dependent protease amplification exerts equivalent biological effects across different metastatic microenvironments. While neutrophil-rich tumors may strongly depend on CTSC-mediated serine protease activation, the relative contribution of CTSC may vary substantially according to immune-cell composition and metastatic niche architecture. Recent systems-level analyses increasingly emphasize that metastatic progression is governed by coordinated protease, stromal, and immune-cell ecosystems rather than by isolated proteases alone [26,27]. Therefore, CTSC should not be interpreted simply as a universal metastasis-promoting enzyme, but rather as a context-dependent upstream regulator whose biological significance depends on the surrounding inflammatory landscape.

Future work should determine which downstream effectors dominate in specific tumor settings and whether CTSC inhibition meaningfully rewires protease network organization in vivo, as these questions will be essential for clarifying its true translational relevance in metastasis.

### 3.2. CTSC, Neutrophils, and NET Formation in Metastasis

Among the currently proposed metastatic mechanisms, the CTSC–neutrophil–NET axis represents one of the most biologically coherent links between protease activation and metastatic niche remodeling. CTSC is required for the activation of major neutrophil serine proteases, including neutrophil elastase, cathepsin G, and proteinase 3 [13]. Because these enzymes participate in neutrophil effector responses, extracellular matrix remodeling, inflammatory amplification, and neutrophil extracellular trap (NET) formation, CTSC is well positioned to influence neutrophil behavior in tumor-associated inflammatory environments [2,5].

More broadly, neutrophil-rich inflammatory programs have been shown to promote metastatic progression in breast cancer and other tumor settings [4,28,29,30,31]. NETs can promote metastasis through several complementary mechanisms, including circulating tumor cell trapping, matrix remodeling, inflammatory signaling, and metastatic niche conditioning [20,32,33]. In this setting, CTSC should be viewed as an upstream enabling factor rather than as a terminal executor of NET-mediated metastasis. By promoting activation of neutrophil serine proteases, CTSC may lower the threshold for NET formation and intensify the inflammatory protease activity that accompanies metastatic niche conditioning [2,5,13].

The relevance of this mechanism is likely to be organ- and context-dependent. Lung metastatic niches are particularly sensitive to neutrophil recruitment, NET-associated tumor cell trapping, and inflammatory reactivation of dormant tumor cells [4,32,33]. Therefore, CTSC-dependent neutrophil protease activation may exert stronger effects in neutrophil-rich metastatic environments than in tumor settings where neutrophilic inflammation is less dominant. This helps explain why the CTSC-NET axis may be especially important in selected metastatic contexts, while not necessarily representing a universal mechanism across all cancers [5,8,19].

Despite its biological plausibility, several unresolved issues remain. It is still unclear whether CTSC promotes NET formation through identical downstream pathways in different tumors, whether tumor-cell-derived and immune-cell-derived CTSC have equivalent roles, and whether CTSC inhibition can suppress NET-associated metastasis without compromising host defense [3,5]. In addition, emerging studies highlight substantial neutrophil heterogeneity within tumor microenvironments, suggesting that not all neutrophil states uniformly promote metastasis [34,35]. Future studies should therefore define which neutrophil subsets, metastatic organs, and inflammatory contexts are most dependent on CTSC-mediated protease activation.

### 3.3. Context Dependency of CTSC-Driven Metastatic Programs

**Figure 2:**
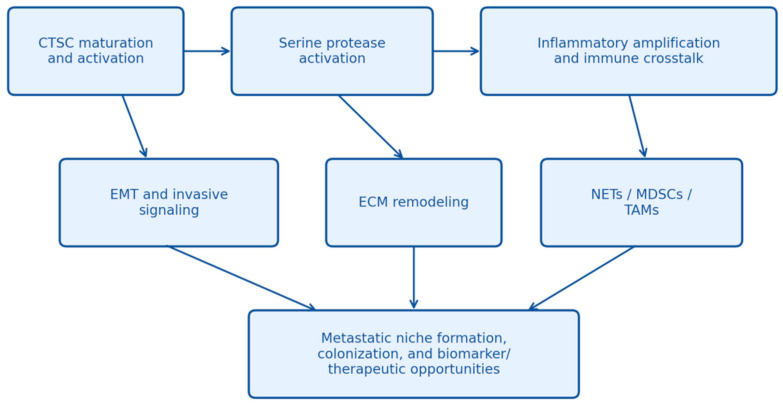
Integrated molecular mechanisms by which CTSC promotes cancer metastasis.

Taken together, the available literature indicates that CTSC should be regarded as a context-dependent regulator of metastatic protease networks rather than as a uniform pan-cancer driver [5,8,19]. Mechanistically, CTSC-related metastatic programs appear to converge on protease network activation, inflammatory amplification, and tumor microenvironment crosstalk, but the dominant downstream pathways differ substantially among tumor types (Figure 2).

In breast cancer, CTSC is most strongly associated with neutrophil recruitment and NET-associated metastatic niche conditioning [7]. In colorectal cancer, the available evidence instead emphasizes CSF1-dependent myeloid remodeling and immune escape involving MDSCs and TAMs [25]. In renal cell carcinoma and non-small-cell lung cancer, current studies more commonly point toward tumor cell-intrinsic migratory signaling or EMT-associated invasive programs [23,36]. In glioma, CTSC is better interpreted as an invasion- or biomarker-associated mediator than as a fully validated metastatic driver [24].

These distinctions suggest that CTSC dependency is shaped by immune cell composition, stromal architecture, tumor-intrinsic signaling, and metastatic organotropism rather than by one conserved pan-cancer mechanism [26,27,37,38,39]. Importantly, CTSC expression alone may not consistently predict metastatic behavior across malignancies. Instead, its biological significance is likely to emerge only when CTSC activity is coupled to permissive downstream protease networks, inflammatory niches, or myeloid cell programs [5,8,19].

Therefore, CTSC is best conceptualized as a context-sensitive upstream amplifier of metastatic protease and inflammatory circuits. This interpretation preserves the therapeutic interest of CTSC while avoiding overstatement. Future mechanistic studies should determine which tumors are truly CTSC-dependent, which downstream proteases or immune cell states mediate this dependency, and whether CTSC inhibition can meaningfully reprogram metastatic protease networks in vivo [5,8,19].

## 4. Tumor-Specific Evidence of CTSC in Metastasis

The tumor types discussed in this section were selected based on the current strength of mechanistic evidence linking CTSC to metastatic progression, inflammatory microenvironment remodeling, or protease network activation. Breast cancer and colorectal cancer currently provide the most biologically integrated evidence, including in vivo metastatic models and tumor–immune microenvironment interactions [7,25]. Hepatocellular carcinoma and renal cell carcinoma provide moderate but still predominantly preclinical evidence centered on invasive signaling pathways [22,36], whereas evidence in NSCLC and glioma remains more exploratory and mechanistically incomplete [23,24]. Importantly, CTSC-related metastatic evidence is still lacking in many other malignancies, including prostate cancer, pancreatic cancer, melanoma, and hematologic cancers. Therefore, the current literature should be interpreted as supporting context-dependent roles of CTSC in selected tumor ecosystems [5,19], rather than a universal pan-cancer metastatic mechanism [8,26,27].

### 4.1. Hepatocellular Carcinoma

Current evidence suggests that CTSC is a plausible but still insufficiently validated mediator of hepatocellular carcinoma (HCC) invasion and metastasis, with the strongest support centered on TNF-α/p38 MAPK-associated inflammatory signaling [22,40,41]. CTSC is upregulated in HCC and has been associated with adverse clinicopathologic features and reduced survival [22]. Functional studies further indicate that CTSC overexpression enhances HCC cell migration and invasion, whereas CTSC silencing attenuates these phenotypes [22]. Broader work on TNF-α-driven hepatocarcinogenesis also supports the biological plausibility that inflammatory signaling may cooperate with protease network activation during HCC progression [41].

However, the current evidence base remains limited in several respects. First, much of the available CTSC-specific evidence in HCC relies on cell-based migration and invasion assays or correlative clinical analyses, whereas robust in vivo metastasis models directly testing CTSC dependency remain limited [22,40]. Second, available clinical cohorts are not yet sufficient to establish whether CTSC provides independent prognostic or metastatic predictive value beyond established HCC variables, such as tumor stage, vascular invasion, inflammatory status, and liver disease background [40,41]. Third, it remains unresolved whether CTSC functions as an independent metastatic driver or mainly reflects activation of broader TNF-α/p38 MAPK-associated inflammatory programs [22,41].

Therefore, CTSC should currently be interpreted as a candidate amplifier of inflammatory-invasive signaling in HCC rather than as a definitively validated metastatic driver. Future studies should determine whether genetic or pharmacologic CTSC inhibition suppresses intrahepatic dissemination or distant metastasis in orthotopic and immune-competent HCC models, and whether CTSC expression improves risk stratification in large clinically annotated patient cohorts [22,40,41].

### 4.2. Renal Cell Carcinoma

In renal cell carcinoma (RCC), CTSC-related evidence remains biologically plausible but comparatively less mature than that in breast cancer or colorectal cancer [36]. The most direct evidence suggests that CTSC is associated with RCC cell migration and invasion, placing it mainly within a tumor cell-intrinsic invasive program rather than within the neutrophil NET-dominant mechanism described in breast cancer lung metastasis [7,36]. This distinction is important because metastatic RCC is clinically heterogeneous and shaped by angiogenic, immune, and metabolic programs that may not be fully captured by a single protease-centered mechanism [42,43].

The best-characterized regulatory model involves an AKT/miR-129-5p-associated pathway. Timosaponin AIII has been reported to suppress RCC cell migration and invasion in association with reduced CTSC expression, while independent evidence supports a tumor-suppressive role for miR-129-5p in clear cell RCC [21,36]. This suggests that CTSC may participate in a broader AKT/miR-129-5p-regulated invasive program rather than functioning as an isolated effector [21,36]. However, this interpretation should remain cautious because miR-129-5p has multiple potential targets, and current data do not yet prove that CTSC is the dominant downstream mediator of this pathway [21].

At present, CTSC is best viewed as a cooperative contributor to RCC invasive behavior rather than as an established RCC-defining metastatic driver. Further studies should clarify whether CTSC knockdown or pharmacologic inhibition suppresses metastasis in orthotopic or metastatic RCC models, whether CTSC adds prognostic value beyond standard clinicopathologic variables, and whether CTSC dependency is restricted to specific molecular or immune subtypes [36,42,43].

### 4.3. Breast Cancer

In breast cancer, the most compelling CTSC-related evidence concerns lung metastatic colonization rather than primary tumor growth [44]. This distinction is mechanistically important because lung metastasis requires tumor cell survival in circulation, arrest within a permissive vascular bed, adaptation to the pulmonary immune niche, and escape from immune surveillance. CTSC expression is elevated in breast cancer lung metastases, and increased CTSC levels in primary tumors have been associated with a higher propensity for lung metastasis and poorer outcomes. In preclinical models, CTSC-targeting interventions reduced spontaneous lung metastasis, supporting a metastasis-enabling rather than a primary growth-promoting role [7].

**Figure 3:**
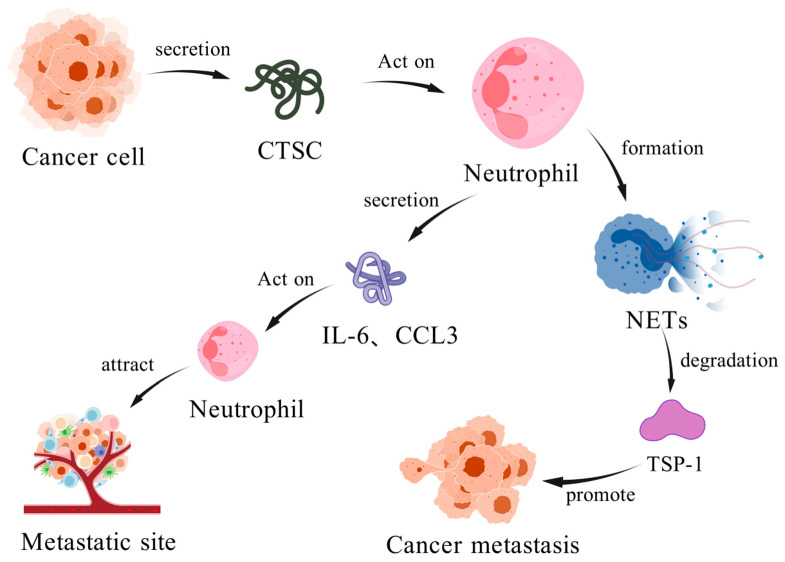
Mechanisms by which CTSC promotes breast cancer metastasis.

The breast cancer model provides the clearest tumor-specific evidence that CTSC can function as a tumor–immune crosstalk molecule. Tumor-derived CTSC has been shown to promote neutrophil recruitment and activation in the lung metastatic niche, thereby generating a feed-forward inflammatory loop favorable for metastatic seeding and colonization [7] (Figure 3). Mechanistically, this process has been linked to activation of neutrophil serine proteases and downstream inflammatory signaling, including the PR3-IL-1β-NF-κB axis, which may amplify cytokine release, neutrophil infiltration, and local inflammatory remodeling [7].

Compared with other tumor types discussed in this review, breast cancer currently provides relatively strong evidence connecting CTSC to an organ-specific metastatic phenotype. Importantly, the reported effects are not limited to CTSC expression as a correlative marker, but include functional perturbation studies showing reduced lung metastasis after CTSC-targeting interventions [7]. These data support the view that CTSC may contribute to breast cancer metastasis by shaping the pulmonary immune microenvironment rather than by simply increasing intrinsic tumor cell proliferation.

From a translational perspective, breast cancer is currently one of the most relevant settings in which CTSC could be explored as a metastasis-related biomarker or therapeutic target. However, biomarker development should not rely on CTSC tissue expression alone. More informative strategies may require combined assessment of CTSC expression, CTSC activity, neutrophil abundance, NET-associated markers, and lung metastasis risk [7,32,45]. This is particularly important because CTSC may identify a neutrophil-rich, inflammation-dependent metastatic phenotype rather than all breast cancers uniformly.

Several uncertainties still limit immediate clinical translation. Most mechanistic evidence derives from preclinical or neutrophil-rich metastatic models, and it remains unclear whether equivalent CTSC dependency exists across luminal, HER2-positive, and triple-negative breast cancer or across different metastatic organs [44,46,47]. In addition, the relative contribution of tumor cell-derived, neutrophil-derived, and stromal CTSC has not been fully clarified [7]. Therefore, CTSC should currently be interpreted as a context-dependent regulator of breast cancer lung metastatic niche formation rather than as a universal breast cancer metastatic driver.

### 4.4. Colorectal Cancer

In colorectal cancer, CTSC-specific evidence is focused mainly on metastasis-associated immune escape rather than on broad tumor-intrinsic proliferation or EMT signaling [25]. This makes CRC an important counterpoint to RCC and NSCLC, where available CTSC-related evidence more strongly emphasizes cell-intrinsic migratory signaling or EMT-associated invasion [23,36]. Thus, the major proposed role of CTSC in CRC is not simply to increase intrinsic invasiveness, but to remodel the immune microenvironment in a way that permits metastatic progression [25,48].

The central mechanism reported to date is a CTSC-CSF1-myeloid axis. CTSC is upregulated in CRC tissues, and experimental data indicate that it promotes metastasis in immunocompetent settings by increasing CSF1 expression and facilitating the accumulation of MDSCs and TAMs within the tumor microenvironment [25]. This mechanism is noteworthy because it places CTSC upstream of a suppressive myeloid circuit rather than downstream of generalized proteolysis, thereby linking protease biology to immune evasion [25]. In this respect, CTSC may serve as a bridge between tumor cell-derived signaling and immune microenvironmental remodeling.

This immune-centered mechanism has potential biomarker implications. If CTSC primarily reflects or drives CSF1-associated myeloid remodeling, then CTSC expression alone may be less informative than combined assessment of CTSC, CSF1, TAM/MDSC abundance, and immune-checkpoint context [25,48]. Such a combined biomarker strategy may help distinguish CRC patients with CTSC-associated immunosuppressive niches from those whose metastasis is driven mainly by tumor-cell-intrinsic invasive programs [25,48]. This distinction is important because CSF1R-directed macrophage reprogramming has been shown to potentiate anti-PD-1 efficacy in colorectal cancer models, supporting the therapeutic relevance of myeloid cell remodeling [48].

However, several gaps limit immediate translation. It is not yet clear whether CTSC-driven CSF1 induction is restricted to specific CRC molecular subtypes, whether it differs between primary tumors and liver or lung metastases, or whether CTSC expression predicts response to immune checkpoint blockade [25,48]. Moreover, it remains unclear whether CTSC inhibition alone is sufficient to reverse established immunosuppressive niches or whether it would need to be combined with CSF1R blockade, checkpoint inhibition, or other microenvironment-directed approaches [25,48]. Taken together, current CRC studies support CTSC as a promising regulator of metastasis-associated immune escape, but not yet as a validated clinical biomarker or stand-alone therapeutic target.

### 4.5. Non-Small-Cell Lung Cancer

**Figure 4:**
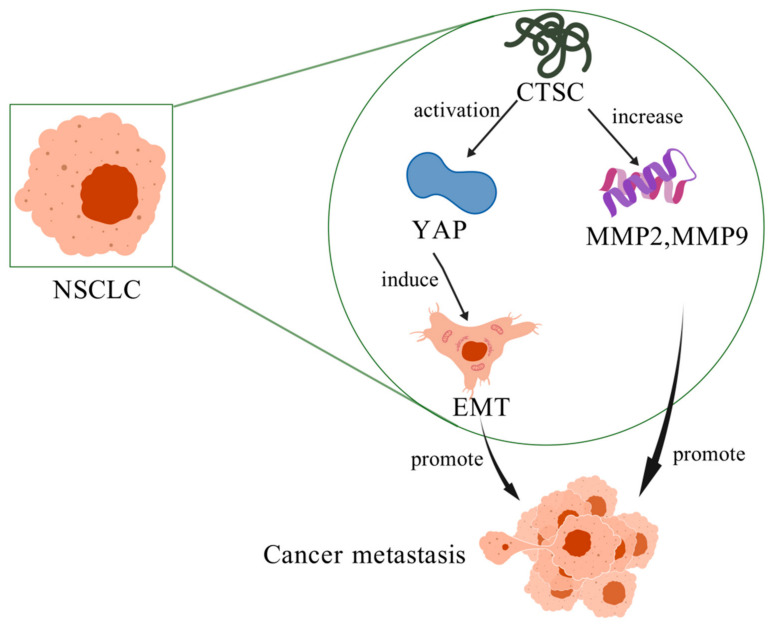
Mechanism by which CTSC promotes NSCLC metastasis.

In non-small-cell lung cancer (NSCLC), available CTSC-specific data mainly support a role in EMT-associated invasion, but validation remains largely preclinical [23]. CTSC overexpression has been reported to promote metastatic behavior by activating YAP-associated EMT programs and by increasing MMP2 and MMP9 expression [23] (Figure 4). Because EMT-associated invasion and metastatic dissemination are major determinants of NSCLC progression and therapeutic resistance, this mechanism is biologically plausible [49,50].

Nevertheless, the strength of evidence remains limited. The YAP/EMT/MMP2/MMP9 axis provides a coherent mechanistic model linking CTSC to tumor cell motility and extracellular matrix remodeling, but current studies do not yet establish whether CTSC is required for NSCLC metastasis in clinically relevant in vivo models [23]. In particular, it remains unclear whether CTSC inhibition can reduce metastatic colonization, whether CTSC acts upstream of YAP activation or in parallel with other EMT drivers, and whether CTSC expression adds prognostic value beyond established molecular and clinical NSCLC markers [23,49,50].

From a translational standpoint, NSCLC should therefore be considered an exploratory setting for CTSC-targeted strategies. CTSC may be most relevant in tumors showing concurrent CTSC upregulation, YAP activation, EMT features, and MMP2/MMP9-associated matrix remodeling [23,50]. However, without in vivo validation and biomarker-defined patient stratification, it remains premature to infer that CTSC inhibition would produce clinically meaningful antimetastatic benefit in NSCLC [23,49,50].

CTSC may induce EMT in NSCLC cells by activating YAP signaling. In addition, CTSC overexpression upregulates MMP2 and MMP9, both of which may contribute to the metastatic potential of NSCLC [23].

### 4.6. Glioma

Current evidence suggests that CTSC in glioma is best viewed as an emerging invasion- or resistance-associated biomarker rather than a fully validated therapeutic target [51]. CTSC is upregulated in glioma and has been linked to invasive behavior through the STAT3/SERPINA3 axis [24]. Additional studies suggest potential biomarker value, associations with radioresistance, and intratumoral heterogeneity [51,52,53]. These observations support a possible role for CTSC in aggressive glioma biology, but the current evidence remains largely preclinical, correlative, or bioinformatic [51,52,53].

A key conceptual issue is that glioma is not a typical distant-metastasis-driven cancer. Unlike epithelial malignancies that frequently disseminate to distant organs, glioma progression is dominated by diffuse local invasion, treatment resistance, intratumoral heterogeneity, and recurrence within the central nervous system [51,52,53]. Therefore, CTSC should not be framed in glioma primarily as a classical distant-metastasis driver. Instead, its relevance may lie in promoting invasive growth, inflammatory or proteolytic remodeling, treatment-resistant phenotypes, or biomarker-defined aggressive states [24,51,52,53].

The STAT3/SERPINA3 axis provides one plausible mechanistic link between CTSC and glioma invasiveness, but causal validation in clinically relevant glioma models remains insufficient [24]. Moreover, the association between cathepsin biology and radioresistance suggests possible therapeutic relevance, yet it remains unclear whether CTSC itself is a functional mediator of resistance or mainly part of a broader lysosomal/protease stress response program [52]. Intratumoral heterogeneity further complicates clinical translation because CTSC expression may vary across tumor regions, cell states, and immune microenvironmental compartments [51,52,53].

At present, CTSC should therefore be positioned as a potential invasion/resistance-associated biomarker in glioma rather than as an established actionable target. Future studies should integrate single-cell analysis, spatial profiling, functional perturbation, and therapy response models to determine whether CTSC contributes directly to glioma invasion or radioresistance, or whether it primarily reflects broader inflammatory and treatment-resistant tumor states [51,52,53].

## 5. Translational Relevance of CTSC: Biomarker Potential and Therapeutic Targeting

### 5.1. Biomarker Potential

A major challenge in developing CTSC as a cancer biomarker is that CTSC may need to be assessed at multiple biological levels rather than as a single static expression marker. Protease-landscape studies indicate that protease dysregulation in cancer frequently reflects coordinated network remodeling rather than isolated single-enzyme overexpression [8]. Broad reviews of cysteine cathepsins likewise emphasize that biomarker value depends on disease context, subcellular localization, extracellular activity, and integration with established clinicopathologic variables [19]. Recent pan-cancer analyses further suggest that CTSC expression is associated with immune infiltration, inflammatory signaling, and heterogeneous prognostic patterns across malignancies, supporting the idea that CTSC-related biomarker value is context dependent rather than universally transferable across tumor types [54].

Accordingly, future biomarker studies should distinguish between tissue-level prognostic association and actionable patient stratification. Immunohistochemical detection of CTSC in primary tumors may not fully capture metastatic relevance if the dominant biology arises from secreted CTSC, immune cell-derived CTSC, lysosomal maturation state, or downstream neutrophil serine protease activation [3,5,10]. Therefore, CTSC expression should ideally be interpreted together with cellular source, maturation state, lysosomal or granule-associated localization, and downstream protease activity [3,5,8]. Neutrophil abundance and NET-associated markers may provide additional context in neutrophil-rich metastatic niches [4,29,30,31]. This integrated approach may better identify tumors in which CTSC is functionally connected to metastatic inflammatory protease networks.

The biomarker implications are also likely to differ by tumor type (Table 1). In breast cancer, CTSC may be most informative when combined with lung metastasis risk, neutrophil infiltration, NET-associated markers, and inflammatory protease activity [7,32,45]. In colorectal cancer, CTSC may be more useful when interpreted together with CSF1 expression, TAM/MDSC accumulation, and immune checkpoint context [25,48]. In HCC, RCC, NSCLC, and glioma, current evidence remains less mature, so CTSC should be treated as a hypothesis-generating biomarker unless validated in larger cohorts and functional models [22,23,24,36,51,52,53].

An important limitation is that CTSC expression alone may not distinguish whether CTSC is a driver, a downstream inflammatory marker, or a surrogate of immune cell infiltration [5,54]. This distinction is crucial for clinical translation because a biomarker used for prognosis may not necessarily identify patients who would benefit from CTSC inhibition [5,54]. Future studies should therefore test whether CTSC improves predictive accuracy beyond standard clinical variables and whether CTSC-linked protease signatures identify patients with targetable inflammatory or myeloid-rich metastatic niches [8,19,54].

### 5.2. Therapeutic Targeting and Translational Opportunities

Therapeutically, CTSC is attractive because it occupies an upstream position in immune-cell serine-protease activation and may therefore modulate multiple downstream inflammatory and proteolytic effectors simultaneously [3,5,10]. In principle, CTSC inhibition could attenuate neutrophil elastase-, cathepsin G-, and proteinase 3-associated inflammatory remodeling, NET-associated metastatic niche formation, and myeloid-linked immune crosstalk [2,3,4,5,7]. This upstream positioning distinguishes CTSC from terminal matrix-degrading proteases and provides a rationale for targeting CTSC in selected inflammatory or neutrophil-rich tumor contexts [5,8].

Current DPP-1/CTSC inhibitors, including brensocatib and BI 1291583, have demonstrated pharmacologic feasibility in inflammatory airway diseases, providing proof of principle that CTSC can be inhibited in humans [55,56]. This experience lowers the translational barrier for oncology because druggability, target engagement, and systemic administration of CTSC/DPP-1 inhibitors are no longer purely theoretical [55,56]. However, evidence supporting CTSC inhibition in cancer remains much less mature than evidence in inflammatory lung disease, and there is currently no robust clinical demonstration that pharmacologic CTSC blockade can suppress metastasis in patients [5,55,56].

The therapeutic opportunity is therefore most plausible in biologically selected settings rather than across all cancers. In breast cancer lung metastasis, CTSC inhibition may be most relevant in tumors with neutrophil-rich inflammatory niches, NET-associated metastatic remodeling, and CTSC-dependent pulmonary colonization [7,28,32,45]. In colorectal cancer, CTSC-targeted approaches may be more rational when linked to CSF1-associated myeloid accumulation, TAM/MDSC-mediated immune escape, or combination strategies involving macrophage reprogramming and immune checkpoint blockade [25,48]. In HCC, RCC, and NSCLC, therapeutic translation remains exploratory because direct evidence for CTSC-dependent metastasis or treatment response is still incomplete [22,23,36]. In glioma, CTSC is better viewed as an invasion- or resistance-associated biomarker rather than a validated therapeutic target [24,51,52,53]. Precision-delivery and nanomedicine approaches may also provide design principles for limiting systemic immune suppression while enhancing microenvironment-directed CTSC modulation [57,58,59].

CTSC inhibition is unlikely to function as a universal monotherapy. Because metastasis is shaped by tumor cells, stromal cells, immune cells, extracellular matrix remodeling, and organ-specific niche biology, blocking CTSC alone may be insufficient in tumors where downstream protease networks are redundant or not CTSC-dependent [5,8,26,27]. More realistic strategies may involve combining CTSC-targeted therapy with immune checkpoint blockade, NET-directed interventions, CSF1R/macrophage-targeted therapy, anti-inflammatory approaches, or other microenvironment-directed treatments [25,28,48,60,61]. Such combinations should be guided by the dominant biology of each tumor context rather than applied uniformly.

Safety is another central issue. CTSC has physiological roles in activating neutrophil, mast cell, and cytotoxic lymphocyte granule proteases, so sustained systemic inhibition could potentially impair antimicrobial defense, alter immune effector functions, or modify inflammatory responses beyond the tumor microenvironment [2,3,5]. Although clinical experience with DPP-1 inhibitors in airway disease provides important safety information, oncology patients may have additional vulnerabilities, including immunosuppression, chemotherapy exposure, infection risk, and advanced disease burden [55,56]. Therefore, future cancer-directed CTSC inhibition will require careful dose selection, treatment duration control, infection monitoring, and patient stratification [2,3,5,55,56].

Patient selection will be essential for reducing risk and improving the likelihood of therapeutic benefit. Because CTSC is unlikely to be a uniform pan-cancer dependency, future trials should prioritize patients with evidence of CTSC activation, neutrophil or myeloid enrichment, NET-associated biology, CSF1-linked immune remodeling, or protease-network activation [4,5,8,25,29,30,31,48,54]. Without such stratification, negative clinical results could reflect biological heterogeneity rather than true failure of CTSC as a target [5,54].

Overall, CTSC is best considered a druggable but incompletely validated metastasis-related target. Its therapeutic value will depend on identifying tumor contexts in which CTSC is functionally required, determining whether CTSC inhibition can rewire downstream protease and immune networks, and balancing antimetastatic benefit against host defense risks [2,3,4,5,8,55,56]. Therefore, the most defensible translational direction is biomarker-guided and mechanism-informed development in selected inflammatory, neutrophil-rich, or myeloid-remodeled tumor settings [5,25,48,54].

### 5.3. Nanomedicine-Informed CTSC Targeting: Opportunities and Current Evidence Gaps

Although CTSC-specific nanomedicine studies in cancer remain scarce, recent advances in cancer nanomedicine provide a useful conceptual framework for improving the delivery, selectivity, and safety of CTSC-directed strategies. Nanoparticle-based delivery systems are increasingly being explored to improve tumor selectivity, reduce systemic toxicity, and modulate the biological and immunological tumor microenvironment [57]. In this context, the relevance of nanomedicine to CTSC should currently be interpreted as a translational opportunity rather than as an established CTSC-specific therapeutic platform.

This concept may be especially relevant in inflammatory and myeloid-rich metastatic settings. Contemporary nanomedicine approaches can preferentially accumulate within tumor-associated inflammatory tissues through enhanced permeability, phagocyte-mediated uptake, local retention, or ligand-directed targeting [57,58,59]. In principle, such strategies could improve delivery of CTSC inhibitors to NET-rich metastatic niches, tumor-associated myeloid cells, or protease-active stromal regions while reducing broad systemic protease suppression. However, these delivery concepts have not yet been directly validated for CTSC inhibitors in metastatic cancer models.

Nanomedicine-based modulation of immune and inflammatory components of the tumor microenvironment is increasingly being investigated as a strategy to improve cancer therapy [57,58,59]. This is relevant to CTSC because CTSC acts upstream of neutrophil serine protease activation and NET formation, two processes closely connected to metastatic niche remodeling [5,7]. Therefore, CTSC-directed nanotherapeutics could theoretically be combined with NET-targeting strategies, including DNase-based therapy, PAD4 inhibition, chemokine-axis blockade, or immune checkpoint inhibition [60,61]. Nevertheless, these possibilities remain speculative because direct evidence for CTSC-loaded, CTSC-selective, or CTSC-responsive nanotherapeutics in cancer is currently limited.

Accordingly, the immediate research priority is not to present CTSC nanomedicine as an established treatment strategy, but to test whether precision delivery systems can improve the therapeutic index of CTSC inhibition in appropriate inflammatory metastatic contexts [5,57,58,59]. Future studies should evaluate nanoparticle compatibility with CTSC inhibitors and tumor- or myeloid-cell delivery specificity [57,58,59], effects on NET-associated metastatic niches [7,60,61], and potential impairment of antimicrobial host defense [2,3,5]. Such work will be necessary before CTSC-directed nanomedicine can be advanced from a biologically plausible concept to a validated translational strategy.

### 5.4. Integrated Microenvironmental Framework for CTSC Translation

Recent NET and tumor microenvironment studies support an integrated translational framework in which CTSC is positioned as one regulatory node within broader inflammatory and proteolytic ecosystems rather than as an isolated metastatic driver [4,26,27,29,30,31]. In this framework, CTSC-related therapeutic relevance is most likely to emerge in tumors with neutrophil or myeloid enrichment, active protease network remodeling, NET-associated niche conditioning, or immune microenvironmental dependence [4,5,7,32,33]. Therefore, future translational studies should integrate CTSC expression with protease-network activity, NET-associated markers, immune cell states, and organ-specific metastatic niche features when evaluating CTSC as a biomarker or therapeutic target [4,8,54].

## 6. Limitations, Future Directions, and Conclusions

Current evidence supports CTSC as a promising but incompletely validated regulator of cancer metastasis at the intersection of protease network activation, inflammatory amplification, and metastatic niche remodeling. Unlike many degradative cathepsins, CTSC appears to act mainly as an upstream activator of immune cell serine proteases, allowing it to influence multiple downstream inflammatory and proteolytic processes simultaneously [3,5,10]. However, the biological and translational relevance of this upstream position is highly context dependent and should not be generalized across all tumor types.

First, future studies should define the tumor settings in which CTSC is functionally required rather than merely overexpressed. The strongest current evidence supports a role for CTSC in breast cancer lung metastasis and colorectal cancer immune microenvironment remodeling, whereas evidence in hepatocellular carcinoma, renal cell carcinoma, non-small-cell lung cancer, and glioma remains more preclinical, correlative, or mechanistically incomplete [7,22,23,24,25,36]. Robust in vivo models, patient-derived systems, and clinically annotated cohorts will be needed to determine whether CTSC inhibition directly suppresses metastatic progression in each tumor context.

Second, biomarker development should move beyond single-marker CTSC expression. Future strategies should integrate CTSC expression with maturation state, cellular source, downstream protease activity, neutrophil or myeloid cell abundance, NET-associated markers, and metastatic organotropism [4,5,8,19,29,30,31,54]. Such integrated biomarker frameworks may better identify patients whose tumors are truly dependent on CTSC-associated inflammatory protease networks.

Third, CTSC-targeted therapy is unlikely to function as a universal monotherapy. More plausible translational strategies may involve mechanism-informed combinations with immunotherapy, NET-directed interventions, macrophage or myeloid cell reprogramming, or microenvironment-targeted therapy [48,60,61]. Because CTSC also contributes to host defense and immune cell protease homeostasis, future therapeutic development should carefully balance antimetastatic efficacy with the risk of excessive immune suppression [2,3,5].

Finally, future research should place CTSC within systems-level models of metastatic ecology. Rather than treating CTSC as a single dominant metastatic switch, studies should evaluate how CTSC interacts with tumor cells, neutrophils, macrophages, extracellular matrix remodeling, organ-specific niches, and broader protease networks [8,26,27,38,39]. This framework may clarify when CTSC is a true therapeutic vulnerability, when it is mainly a biomarker of inflammatory tumor ecology, and how CTSC-targeted strategies can be rationally incorporated into precision oncology.

Overall, current evidence supports CTSC as a promising but incompletely validated target at the intersection of protease activation, inflammatory amplification, and metastatic niche remodeling. Its ultimate translational value will depend on whether context-specific CTSC dependency can be integrated into biomarker-guided and mechanism-informed therapeutic strategies.

## Figures and Tables

**Table 1:** Cancer-specific evidence for CTSC in metastasis: mechanisms, evidence level, and translational relevance.

Tumor Type	Major CTSC-Linked Mechanism	Evidence Base	Metastatic Phenotype	Biomarker Promise	Key Limitation	Key References
HCC	TNF-α/p38 MAPK-associated invasive signaling	Cell-based studies + limited clinical correlation	Migration/invasion; metastasis not fully validated in vivo	Possible prognostic marker	Mostly preclinical; limited in vivo metastasis validation; causality not fully established	[22,40,41]
RCC	AKT/miR-129-5p-regulated CTSC expression	Mainly cell-based	Migration/invasion	Potential aggressive disease marker	Insufficient in vivo and prognostic validation	[21,36,42,43]
Breast cancer	Neutrophil crosstalk, PR3-IL-1β-NF-κB axis, NETs	Cell + animal + clinical correlation	Lung metastasis	Strongest current biomarker rationale	Subtype generalizability and clinical validation remain limited	[7,28,32,44,45,46,47]
CRC	CSF1-mediated MDSC/TAM recruitment and immune escape	Cell + animal + tissue studies	Metastasis-associated immune escape/myeloid remodeling	Promising in immune context settings; may require combined CTSC-CSF1-myeloid assessment	Patient selection criteria remain unclear; predictive value for immunotherapy not established	[25,48]
NSCLC	Hippo/YAP activation, EMT, MMP2/MMP9 upregulation	Mainly preclinical	Migration/invasion	Preliminary	Clinical sensitivity and specificity remain undefined	[23,49,50]
Glioma	STAT3/SERPINA3 axis and TIME modulation	Preclinical + bioinformatic/clinical association	Invasiveness/treatment resistance	Potential prognostic marker	Mechanistic depth and therapeutic validation are limited	[24,51,52,53]

## Data Availability

No new data were created or analyzed in this study. Data sharing is not applicable to this article.
